# Measuring General Health Literacy in Haitian Immigrant Adults: Validation of the HLS_19_-Q12 Instrument in Haitian Creole

**DOI:** 10.3390/ijerph23050554

**Published:** 2026-04-25

**Authors:** Maurice J. Chery, Eric C. Brown, Arsham Alamian, Jovanka Ravix, Sandy St. Hilaire, Aisha Severe, Lauren Smith, Reginald Fils-Aime, Mary Clisbee, Rimsky Denis, Samara Perez, Justin J. Sanders, Donaldson Conserve, Judite Blanc, Joseph Bernard, Patricia Moreno, Matthew P. Schlumbrecht, Sophia H. L. George

**Affiliations:** 1 Department of Public Health Sciences, University of Miami Miller School of Medicine, Miami, FL 33136, USA; ricbrown@miami.edu (E.C.B.); acs533@miami.edu (A.S.); patricia.moreno@miami.edu (P.M.); 2 Sylvester Comprehensive Cancer Center, University of Miami Miller School of Medicine, Miami, FL 33136, USA; j.ravix@med.miami.edu (J.R.); mschlumbrecht@med.miami.edu (M.P.S.); sophia.george@med.miami.edu (S.H.L.G.); 3 School of Nursing and Health Studies, University of Miami, Coral Gables, FL 33146, USA; arsham.alamian@miami.edu; 4 Department of Obstetrics, Gynecology, and Reproductive Sciences, University of Miami Miller School of Medicine, Miami, FL 33136, USA; sxs3292@med.miami.edu (S.S.H.); las501@miami.edu (L.S.); 5 Research Department, Hôpital Universitaire de Mirebalais, Zanmi Lasante, Mirebalais HT 5210, Haiti; rfilsaime@pih.org (R.F.-A.); mclisbee@pih.org (M.C.); 6 Center for Haitian Studies, Miami, FL 33138, USA; rimskydenis@gmail.com; 7 Department of Oncology, Research Institute of the McGill University Health Centre, Montreal, QC H4A 3T2, Canada; samara.perez@mcgill.ca; 8 Department of Family Medicine, Research Institute of the McGill University Health Centre, Montreal, QC H3S 1Z1, Canada; justin.sanders@mcgill.ca; 9 Department of Prevention and Community Health, Milken Institute School of Public Health, The George Washington University, Washington, DC 20051, USA; dconservejr@email.gwu.edu; 10 Department of Psychiatry and Behavioral Sciences, University of Miami Miller School of Medicine, Miami, FL 33136, USA; juditeblanc@miami.edu; 11 Clinique du Cancer Saint-Francois de Sales, Port-au-Prince HT 6110, Haiti; josephbernardjr.ht@gmail.com

**Keywords:** Haitian immigrants, health literacy, Haitian Creole, psychometric validation, HLS_19_-Q12

## Abstract

**Highlights:**

**Public health relevance—How does this work relate to a public health issue?**

- Haitian immigrants face health literacy challenges linked to language barriers, recent migration, and difficulty navigating the U.S. healthcare system.
- This study addresses the lack of a psychometrically validated general health literacy instrument in Haitian Creole, a major gap for equitable public health measurement.

**Public health significance—Why is this work of significance to public health?**

- The Haitian Creole HLS_19_-Q12 showed strong reliability and validity, supporting its use as a sound measure of general health literacy in this underserved population.
- Findings revealed substantial difficulty with health system navigation, treatment decision-making, and understanding emergency and mental health information.

**Public health implications—What are the key implications or messages for practitioners, policy makers and/or researchers in public health?**

- The HLS_19_-Q12-HC can be used in research, community assessment, and intervention evaluation to identify health literacy needs among Haitian Creole-speaking populations.
- Results support the need for language-concordant communication, culturally responsive health education, and patient navigation strategies to reduce inequities in care access and use.

**Abstract:**

Background: Haitian immigrants in the United States face health literacy challenges related to recent migration, language discordance, and unfamiliar healthcare systems, yet no general health literacy instrument has been psychometrically validated in Haitian Creole. This study translated, culturally adapted, and evaluated the Haitian Creole HLS_19_-Q12 (HLS_19_-Q12-HC). Methods: Haitian Creole-speaking adults without cancer diagnoses in South Florida (*n* = 168) completed the HLS_19_-Q12-HC and the Haitian Creole Brief Health Literacy Screen. Translation included forward–backward procedures, expert review, and cognitive interviews (*n* = 7). Psychometric evaluation used confirmatory factor analysis, reliability testing, and assessment of convergent and known-groups validity. Results: Cognitive interviews supported clarity and cultural appropriateness with minor refinements. Reliability was excellent (ω = 0.949; α = 0.944; AVE = 0.584). The unidimensional model showed good fit (CFI = 0.951; TLI = 0.944; RMSEA = 0.065; SRMR = 0.048), whereas multi-factor models showed limited discriminant validity. Convergent and known-groups validity were supported. Using provisional European-derived cutpoints, 70.2% of participants were classified as having inadequate or problematic health literacy. Conclusions: The HLS19-Q12-HC showed evidence of reliability and validity as a unidimensional measure of general health literacy and may support research, needs assessment, and culturally responsive interventions for Haitian Creole-speaking populations. Findings should be interpreted in light of the convenience sample from South Florida and the predominantly female composition of the cohort.

## 1. Introduction

Health literacy, or the extent to which individuals can obtain, process, understand, appraise, and apply health information needed to make appropriate health decisions [1], is a critical determinant of health outcomes and health equity [2,3]. Conceptually, health literacy extends beyond individual reading and comprehension skills to encompass the interaction between individual capacities and the complexity, accessibility, and cultural responsiveness of health systems [4]. This socioecological perspective recognizes that health literacy is not a static individual trait but rather emerges from the dynamic relationship between people’s knowledge, skills, and motivations and the demands placed on them by healthcare environments. Limited health literacy has been associated with poorer health outcomes, increased hospitalization, reduced adherence to treatment recommendations, and higher healthcare costs [5]. Consequently, accurate population-level measurement of health literacy is fundamental for identifying vulnerable groups, monitoring disparities, informing targeted interventions, and evaluating policy initiatives aimed at advancing health equity.

Immigrant populations often experience compounded health literacy challenges, as they must navigate unfamiliar healthcare systems while confronting structural barriers, cultural differences, and linguistic obstacles [6]. Haitian immigrants in the United States, and particularly those in South Florida, which is home to one of the largest Haitian diaspora communities globally, face socioeconomic marginalization, limited access to culturally tailored services, and constrained English proficiency, all of which may impede their effective use of health information [7,8]. Among limited-English-proficient adults in the United States, low health literacy is common; a study found that 68.3% of limited-English-proficient adults had low health literacy compared with 30.1% of English-proficient adults [9], underscoring the amplified vulnerability of recently arrived immigrant communities. Recent Haitian immigrants often arrive following economic instability, political upheaval, and natural disasters in Haiti, with many experiencing disrupted education, limited prior healthcare access, and trauma that may further complicate health system navigation in the United States. These contextual factors underscore the need for culturally and linguistically appropriate health literacy assessment tools that can accurately characterize the challenges faced by this population and guide the development of responsive interventions.

Language represents a central methodological challenge in assessing health literacy within Haitian communities. Although Haiti has two official languages, French and Haitian Creole, the latter is the primary language spoken by virtually the entire population. Among Haitian immigrants, especially those from lower socioeconomic or rural backgrounds, Haitian Creole remains dominant. Administering health literacy instruments in English or French risks conflating language proficiency with health literacy, introducing measurement error and potentially biasing estimates. Furthermore, commonly used health literacy frameworks emphasize written, individual-level tasks and may not fully reflect the oral communication patterns and community-based information sharing that are important in Haitian communities [4,10]. Valid assessment requires instruments that are available in Haitian Creole and validated psychometrically within Haitian Creole-speaking contexts.

Several established health literacy instruments exist, but each raises distinct construct-validity and cross-cultural measurement concerns when applied in immigrant populations. The original TOFHLA [11] and REALM [12] primarily assess reading, numeracy, and word-recognition performance in relation to written medical materials and therefore reflect a narrower functional-literacy approach than contemporary multidimensional health literacy frameworks [1,4]. In immigrant populations, these task demands are shaped by language discordance, disrupted schooling, and unfamiliarity with standardized testing formats, introducing construct-irrelevant variance [13] and item- and method-level bias in cross-cultural measurement [14]. By contrast, eHEALS [15] focuses specifically on digital health information skills rather than general health literacy across healthcare, disease prevention, and health promotion contexts. eHEALS also presupposes digital access and skills, embedding a construct-irrelevant dependence that may disadvantage immigrant and low-resource populations affected by the digital divide [16]. For the present study, a brief multidimensional instrument suitable for administration in Haitian Creole was needed. A measure designed for population-level assessment was especially important given the study’s goal of supporting disparities research and future intervention development. The HLS_19_-Q12 was therefore selected because it is brief, multidimensional, and designed for population-level use across languages and settings [17,18].

The HLS_19_-Q12 has been adapted and evaluated in multiple languages, but these validations have highlighted several recurring methodological challenges that justify population-specific psychometric evaluation. First, factor-structure variability has been reported across settings: although the instrument was conceptualized across three domains, Chinese [19], Turkish [20], and Swedish [21] validations have often shown very high inter-factor correlations and empirical convergence toward a general health literacy factor, raising questions about the discriminant validity of the theorized subdomains in applied monitoring contexts. Second, cross-cultural validity cannot be assumed across linguistic and sociocultural settings, particularly when the instrument is used in populations with different healthcare systems, communication practices, and norms regarding information-seeking and decision-making [4,22]. Third, measurement invariance, a prerequisite for valid cross-group score comparison, has not been consistently examined or established across language versions, leaving open the possibility that observed differences reflect measurement artifacts rather than true health literacy differences [23]. Taken together, these findings suggest that psychometric adequacy in one setting should not be assumed to transfer automatically to another. To our knowledge, no validated Haitian Creole version of the HLS_19_-Q12 exists, and no general health literacy instrument has been psychometrically validated for Haitian Creole-speaking populations.

Cross-cultural adaptation requires careful evaluation of linguistic clarity, conceptual equivalence, and psychometric performance [24]. Translation alone does not establish whether an instrument functions reliably and validly in a new cultural context. Thus, this study contributes beyond linguistic translation by evaluating whether the instrument retains appropriate psychometric performance in this population. Without such validation, health literacy estimates may be inaccurate, potentially obscuring disparities and misinforming intervention effectiveness. The present study translated, culturally adapted, and psychometrically validated the Haitian Creole version of the HLS_19_-Q12 (HLS_19_-Q12-HC) among Haitian immigrants in South Florida.

## 2. Materials and Methods

### 2.1. The Ethics

The study was approved by the University of Miami Institutional Review Board, was conducted within the Cancer Health Disparities Registry (CHDR; IRB no. 20160983), and was conducted in accordance with the Declaration of Helsinki [25]. All participants provided written informed consent. Participants received a $50 incentive for participation in the broader CHDR protocol, which included a survey lasting approximately 90 min as well as blood and saliva collection.

### 2.2. Study Setting, Sample, and Data Collection

The CHDR is an ongoing study examining biological, cultural, and psychosocial drivers of breast cancer survival disparities across Black subpopulations in South Florida. CHDR is a comprehensive registry designed to address the disproportionate burden of breast cancer mortality among Black women in the United States, including African American, Afro-Caribbean, and African immigrant populations. The registry integrates clinical data, biospecimens, and psychosocial surveys to characterize factors contributing to survival disparities and to inform interventions tailored to diverse cultural contexts within the African diaspora.

Within CHDR, the Haitian sub-cohort—Sante Nou Se Avni (“Our Health is Our Future”)—focuses on Haitian Creole-speaking participants. Although CHDR enrolls both healthy individuals and those with cancer diagnoses, the present validation sample was drawn exclusively from healthy community members without cancer diagnoses at the time of enrollment, recruited to establish baseline health literacy profiles. CHDR eligibility criteria include self-identifying as Black, age ≥18 years, and ability to communicate in English, Spanish, French, or Haitian Creole.

Recruitment for the Haitian sub-cohort used complementary clinic-based and community-based strategies. Community-based recruitment at cultural festivals and community centers was intended to reach individuals with varying levels of experience in the U.S. healthcare system, including those who may not have established regular contact, thereby enhancing diversity with respect to healthcare exposure and acculturation. The CHDR was established through voluntary enrollment of individuals introduced to the study by trained research staff in participating clinics and community settings, including community events. Individuals who expressed interest and met eligibility criteria were then enrolled in the registry. Accordingly, the validation sample should be considered a convenience sample rather than a probability-based sample. As of December 2025, approximately 380 participants had enrolled in CHDR; 168 Haitian Creole speakers without cancer diagnoses at enrollment constituted the validation sample, exceeding the commonly recommended a 10:1 participant-to-item ratio for the 12-item scale [26,27]. Data were collected via comprehensive surveys administered in person or online by trained bilingual staff fluent in Haitian Creole. The survey included the HLS_19_-Q12-HC, items assessing cancer causal and prevention beliefs, and psychosocial and demographic measures. Seven participants also were recruited separately for cognitive interviews prior to quantitative validation to ensure translation quality and cultural appropriateness before field deployment.

### 2.3. Instruments

#### 2.3.1. HLS_19_-Q12

The HLS_19_-Q12 is a 12-item self-report questionnaire assessing four health information management constructs (accessing, understanding, appraising, and applying) across three domains (healthcare, disease prevention, and health promotion) [17]. Items were rated on a 4-point Likert scale ranging from 1 (“very difficult”) to 4 (“very easy”), with respondents indicating the ease of completing specific health literacy tasks (e.g., “On a scale from very easy to very difficult, how easy would you say it is to judge the advantages and disadvantages of different treatment options?”). Index scores were computed as the mean of completed items, rescaled from zero to 100, and categorized as inadequate (≤50), problematic (>50–66.67), sufficient (>66.67–83.33), or excellent (>83.33) using established HLS cutpoints for provisional comparison with prior studies.

#### 2.3.2. Brief Health Literacy Screen (BHLS)

The BHLS is a four-item screener assessing confidence with medical forms and understanding health materials [28]. Items asked respondents to rate their confidence in completing medical forms by themselves, how often they needed help reading hospital materials, how often they had problems learning about medical conditions due to difficulty understanding written information, and how confident they were in understanding written health information. Items were rated on five-point Likert scales and reverse-coded where appropriate so that higher scores consistently indicated better health literacy. Total scores (4–20) were categorized as low (4–8), moderate (9–12), or high (13–20) health literacy [29].

#### 2.3.3. Translation and Cultural Adaptation

The BHLS was translated into Haitian Creole (BHLS-HC) using forward–backward translation consistent with the HLS_19_-Q12-HC process. The BHLS-HC demonstrated good reliability in this sample (α = 0.87, ω = 0.88; see Supplementary Table S1). Translation followed M-POHL guidance [17,18]. Two independent forward translations were produced by native Haitian Creole speakers fluent in English (first author [MJC] and certified translator [JB]). A health education expert (RFA) reviewed both and selected optimal wording emphasizing plain language and accessibility. An expert panel (two public health specialists [JKL; ET] and one linguist [YAN], all native Haitian Creole speakers) reviewed the consolidated version and suggested refinements. Back-translation was conducted by a bilingual certified translator (WBM) who was independent of the forward translation process and had no prior knowledge of the instrument. A native English-speaking reviewer (MC) compared the back-translation with the original English version and judged the versions to be conceptually equivalent (Supplementary Table S5).

### 2.4. Content Validity

Cognitive interviews with seven Haitian Creole-speaking participants assessed clarity, comprehensibility, and cultural appropriateness. Participants were recruited to represent diversity in age (18–40 vs. 45–70), sex (five women, two men), and education (two with higher education, three who had completed high school, and two with some high school), ensuring that translation quality could be evaluated across the sociodemographic spectrum of the target population. Interviews used think-aloud protocols in private settings at the Center for Haitian Studies, a trusted community partner organization. *Standardized probing questions included: “Can you tell me in your own words what this question is asking?”; “Was there anything confusing or unclear about the wording?”; “Did anything seem irrelevant or not applicable to your life?”; and “Did the response options make sense?”* Written notes were recorded independently by the first author and a second trained note taker (AS) using a structured documentation protocol that included standardized probing questions and verbatim recording of key participant responses. Notes were compared after each interview to ensure completeness and accuracy. Interviews lasted 15–20 min and were not audio-recorded.

### 2.5. Psychometric Testing and Data Analysis

Psychometric analysis followed Consensus-based Standards for the selection of health Measurement Instruments (COSMIN) [30], using *Mplus* version 8.10 [31], and assessed internal construct validity, external construct validity, and reliability. COSMIN provides internationally recognized standards for evaluating the methodological quality of studies on measurement properties, ensuring rigorous and transparent psychometric assessment. Given the cross-sectional design of this registry-based validation study, participants were assessed at a single time point; therefore, test–retest reliability was not evaluated.

### 2.6. Internal Construct Validity

Confirmatory factor analysis (CFA) compared three models: (1) unidimensional, (2) two-factor (clinical/healthcare navigation vs. health promotion/everyday), and (3) three-factor (HLS-EU domains: healthcare, disease prevention, and health promotion). Testing multiple theoretical structures allowed evaluation of whether the conceptual multidimensionality underlying the HLS_19_-Q12 manifested empirically or whether items functioned as a single general health literacy factor, as supported by prior validations in diverse populations [19,21].

Robust maximum likelihood (MLR) estimation was used due to sparse “very easy” category endorsement (6.8% overall, 3.6–8.3% per item; see Supplementary Table S2). Because the highest response category was sparsely endorsed, we used MLR, which performs well with imbalanced ordinal data. As a sensitivity analysis, we also estimated models using WLSMV estimation. Consistent with prior work, RMSEA was inflated under categorical estimation with sparse categories despite high incremental fit indices [32,33,34]. Sensitivity analyses using WLSMV to assess the robustness of findings to estimator choice are reported in Supplementary Table S6.

Model fit was evaluated using CFI, TLI, RMSEA (with 90% CIs), and SRMR. Acceptable fit criteria included CFI/TLI ≥ 0.90, RMSEA ≤ 0.08, and SRMR ≤ 0.08; good fit criteria included CFI/TLI ≥ 0.95 and RMSEA ≤ 0.06 [32], with ΔCFI < 0.01 indicating that the more parsimonious model is preferred [35]. Factor loadings of ≥0.40 were considered acceptable, ≥0.70 strong, and ≥0.80 very strong [36]. Inter-factor correlations of rs > 0.85 suggested limited discriminant validity [35].

### 2.7. External Construct Validity and Reliability

External construct validity was examined through two complementary approaches: convergent validity and known-groups validity. Convergent validity examined whether the HLS_19_-Q12-HC correlated positively with another measure of the same construct. Convergent validity was assessed in a structural equation modeling (SEM) framework by regressing BHLS-HC total scores (range 4–20; higher scores indicated better health literacy) on the latent HLS_19_-Q12 health literacy factor using maximum likelihood with robust standard errors (MLR), which was appropriate for models with mixed ordinal indicators and continuous outcomes. We hypothesized a moderate-to-strong positive association between the two measures because the two instruments measure related aspects of health literacy, although they differ in scope, with the HLS_19_-Q12 assessing multidimensional health literacy and the BHLS serving as a brief screener. Effect sizes were interpreted using standard thresholds: negligible (0–0.09), weak (0.10–0.39), moderate (0.40–0.69), strong (0.70–0.89), and very strong (0.90–1.0) [37].

Known-groups validity was assessed by testing whether the measure differentiated across education groups that were theoretically expected to vary in health literacy. Within the SEM framework, education was specified as an ordinal predictor of latent health literacy (1 ≤ high school, 2 = high school graduate/GED, 3 = some college/vocational, 4 = college degree or higher). Education was selected as the primary known-groups variable based on prior evidence showing that individuals with higher levels of formal education tend to report higher health literacy [38]. We therefore hypothesized a significant positive association between education level and HLS_19_-Q12-HC latent scores, consistent with evidence from prior HLS validations [38,39]. To complement the SEM analysis and aid interpretation, we also examined descriptive statistics and conducted one-way ANOVA with Tukey HSD post hoc tests across education groups [40,41].

Internal consistency was evaluated using McDonald’s omega (ω) calculated from CFA factor loadings [42]. Additionally, average variance extracted (AVE) was computed to evaluate convergent validity at the construct level, with values ≥0.50 indicating adequate convergent validity. Item–total correlations were also computed to further support internal consistency and are reported alongside item-level descriptive statistics.

## 3. Results

### 3.1. Cognitive Interviews

Seven Haitian Creole-speaking adults completed cognitive interviews. Participants generally understood the instructions, items, and response options as intended, and no content was perceived as confusing, offensive, or culturally inappropriate. Feedback was grouped into four broad categories: comprehension and clarity, terminology accessibility, cultural familiarity of examples, and response option interpretability. Clinical concepts were more easily understood when accompanied by plain-language examples, and participants were more familiar with Pap tests and mammography than with colorectal cancer screening, prompting example substitution in Item 6. Participants also indicated that adding examples such as “stress, depression, or anxiety” made the mental health item more relatable to everyday experience. These findings provided qualitative evidence supporting the translation’s linguistic clarity and cultural appropriateness prior to quantitative psychometric testing.

### 3.2. Psychometric Properties

#### 3.2.1. Participants’ Characteristics

Among 168 Haitian adults (Table 1), median age was 46 years (range 21–87); 142 (84.5%) were women. Median U.S. residence was 2 years (range 1–50), with 115 (68.5%) having resided in the U.S. for 1–2 years, reflecting a sample of predominantly recent arrivals in the early stages of acculturation to the U.S. healthcare system. Education was mixed: 20 (11.9%) had ≤high school education, 35 (20.8%) were high school graduates, 67 (39.9%) had completed some college/vocational training, and 46 (27.4%) had a college degree or higher. Although 67.3% had some post-secondary education, only 27.4% held completed degrees. Most (75.0%) reported household income <$15,000.

Median health literacy score was 52.3 (range 0–100) on the HLS_19_-Q12-HC, with 82 (48.8%) classified as inadequate and 36 (21.4%) as problematic using the European-derived cutpoints. Median BHLS-HC score was 10.0 (range 4–20; *n* = 159; 9 participants had missing data), with 67 (42.1%) classified as having low, 33 (20.8%) moderate, and 59 (37.1%) high health literacy. The distribution across BHLS-HC categories was similar to that observed on the HLS_19_-Q12-HC, with the majority (62.9%) classified as having low or moderate health literacy, providing preliminary evidence of consistency across measurement approaches.

#### 3.2.2. Item-Level Descriptive Results & Reliability

Item-level responses indicated generally low perceived ease (overall mean 2.47 ± 0.74 on 1–4 scale), with 50.3% reporting “*difficult*” or “*very difficult*” (Table 2). The highest difficulty rates were for finding mental health information (63.1%), judging treatment advantages/disadvantages (56.0%), and understanding medical emergency information (56.0%). The lowest difficulty rates were for understanding family/friends’ health advice (39.9%) and finding healthy lifestyle information (44.6%). Using the conventional 15% threshold, no ceiling effects were observed, as endorsement of the “*very easy*” category ranged from 3.6% to 8.3% across items. Endorsement of the “*very difficult*” category ranged from 10.7% to 26.2%. Most items were below the 15% threshold for floor effects; however, Item 3 (judging treatment advantages/disadvantages, 26.2%), Item 5 (finding mental health information, 24.4%), and Item 1 (finding professional help when ill, 22.6%) exceeded it (Supplementary Table S2). Corrected item–total correlations ranged from 0.685 to 0.818, further supporting meaningful contribution of all items to the overall scale. Overall, 70.2% were classified as having inadequate or problematic health literacy. The HLS_19_-Q12-HC demonstrated excellent internal consistency (Table 3): McDonald’s ω = 0.949 and Cronbach’s α = 0.944. Average variance extracted (AVE = 0.584) exceeded the 0.50 threshold, supporting that the items shared substantial common variance with the latent factor.

#### 3.2.3. Construct Validity

The unidimensional model showed good fit (CFI = 0.951, TLI = 0.944, RMSEA = 0.065 [90% CI: 0.044–0.084], SRMR = 0.048; Table 4). Two-factor and three-factor models showed numerically improved fit (2-factor: CFI = 0.978, TLI = 0.974, RMSEA = 0.044; 3-factor: CFI = 0.972, TLI = 0.967, RMSEA = 0.050). Compared with the unidimensional model, ΔCFI values were +0.027 for the 2-factor model and +0.021 for the 3-factor model. Inter-factor correlations were very high (2-factor *r* = 0.91; 3-factor *r* = 0.886–0.942; see Supplementary Table S3), suggesting limited discriminant validity. The unidimensional model was retained as the most parsimonious. All items showed strong-to-excellent standardized loadings (*λ* = 0.690–0.851, all *p*s < 0.001) with *R*^2^ = 0.476–0.724 (Table 5); the strongest loadings were for judging how housing conditions may affect health (λ = 0.851), making health improvement decisions (*λ* = 0.836), and judging information reliability (*λ* = 0.834), whereas the weakest loading was for finding professional help when ill (*λ* = 0.690). Almost all factor loadings exceeded the 0.70 threshold for strong loadings, indicating that each item contributes meaningfully to the measurement of the underlying health literacy construct.

SEM supported construct validity through convergent and known-groups evidence (Table 6). For convergent validity: HLS_19_-Q12-HC and BHLS-HC showed a moderate-to-strong association (*β* = 0.518, *SE* = 0.058, *p* < 0.001, 95% CI [0.405, 0.631]), with 26.8% shared variance, indicating that instruments measured related, but substantially distinct, aspects of health literacy consistent with their differences in scope (comprehensive multidimensional assessment vs. brief functional screener).

Examination of known-groups validity indicated that education strongly predicted health literacy (*β* = 0.463, *SE* = 0.074, *p* < 0.001, 95% CI [0.318, 0.608]), demonstrating that the HLS_19_-Q12-HC successfully detected theoretically expected differences between education groups. This aligned with observed scores: ≤high school *M* = 22.4 (*SD* = 35.3) vs. college degree or higher *M* = 70.8 (*SD* = 31.4), a 48-point difference. ANOVA confirmed significant group differences (*F*(3, 164) = 12.68, *p* < 0.001, *η*^2^ = 0.19), indicating a large effect size. Post hoc comparisons (Tukey HSD) showed all pairwise differences to be significant (*p* ≤ 0.001) except ≤high school vs. high school graduate/GED (*p* = 0.382; see Supplementary Table S4). The largest differences were between ≤high school vs. college degree or higher (−48.4 points, *p* < 0.001, *d* = −1.46) and high school graduate/GED vs. college degree or higher (−38.1 points, *p* < 0.001, *d* = −1.08).

## 4. Discussion

This study translated, culturally adapted, and psychometrically validated the Haitian Creole version of the HLS_19_-Q12 for Haitian Creole-speaking immigrants in South Florida. Using cognitive interviews and COSMIN-guided latent-variable psychometric testing, we found evidence supporting the reliability and validity of the HLS_19_-Q12-HC as a measure of general health literacy in this population, with results consistent with the international HLS_19_ measurement program and other short-form HLS validations [17,43].

Cognitive interviews indicated that respondents across various age and education groups understood instructions, items, and response options as intended, and no items were perceived as culturally inappropriate. Modifications were minor (e.g., plain-language phrasing and culturally familiar examples), aligning with M-POHL guidance that prioritizes functional equivalence over literal translation in cross-cultural adaptation [17,18]. Similar patterns have been reported in other HLS short-form adaptations, where cognitive testing typically supports conceptual retention while prompting small refinements to improve accessibility [20,43].

The HLS_19_-Q12-HC showed excellent internal consistency (ω = 0.949; α = 0.944) and adequate convergent reliability (AVE = 0.584). These reliability estimates are somewhat higher than those reported in several validations of the HLS_19_-Q12/HLS-Q12 family. For example, a recent study using HLS_19_-Q12 reported (α = 0.885) and (ω = 0.877), illustrating that “good-to-acceptable” reliability is common in diverse samples [44]. Other language adaptations have reported similar or somewhat lower reliability; for example, the Turkish validation reported alpha values in the high 0.80s [20]. The higher reliability observed here may reflect (1) the plain-language Haitian Creole translation and anchoring examples improving clarity, and/or (2) greater homogeneity in structural vulnerability (recent arrival, low income, limited system familiarity) producing consistently low perceived ease across items. Importantly, very high reliability does not necessarily indicate redundancy in short instruments; in the original HLS-Q12 development work, a key goal was to create a psychometrically efficient 12-item measure while reducing redundancy relative to longer forms [43]. The corrected item–total correlations (range: 0.685–0.818) further support the coherence of the scale and suggest that even the weakest items remained meaningfully related to the overall construct.

CFA results support strong internal construct validity. Across studies, the HLS_19_-Q12 is often treated as a practical index for general health literacy monitoring and cross-population comparisons, even though it originates from a multidimensional conceptual matrix [17,18]. In our sample, two- and three-factor solutions produced slightly improved global fit indices, but inter-factor correlations were extremely high (*r*s = 0.886–0.942), indicating limited discriminant validity and supporting a parsimonious unidimensional interpretation. This pattern suggests that the proposed subdomains were not empirically distinct in this sample, despite their conceptual relevance. This finding also underscores the importance of population-specific psychometric evaluation in cross-cultural adaptation, as the dimensional structure and comparability of short-form health literacy measures cannot be assumed to transfer automatically across linguistic and sociocultural contexts, particularly when measurement invariance has not yet been established. This pattern is consistent with evidence that short-form HLS instruments frequently behave as a single general factor in applied monitoring contexts, and that domain distinctions may be empirically weak when respondents face shared system-level constraints [21]. Notably, the M-POHL consortium emphasizes that HLS_19_-Q12 is designed for population measurement; the practical objective is often robust estimation of overall health literacy rather than sharply separable subscales [17,18].

External construct validity was supported through convergent and known-groups evidence. The latent HLS_19_-Q12-HC factor was moderately to strongly associated with BHLS-HC scores (*β* = 0.52; *R*^2^ = 0.27). This magnitude is appropriate given differences in scope: the HLS_19_-Q12 captures broader information-management competencies across contexts, whereas brief screeners tend to emphasize confidence with written materials and medical forms. The meaningful but not redundant overlap is expected when comparing comprehensive health literacy measures with brief functional screeners.

Known-groups validity was supported by a clear educational gradient (*β* = 0.463), consistent with evidence from large-scale European measurement and other settings that education is among the most stable correlates of health literacy [39]. Importantly, immigrant-focused literature also emphasizes that education obtained outside the host country may not translate directly into system navigation competence when language discordance and unfamiliar institutional procedures are present, helping explain why a sample may show both strong within-sample education gradients and an overall downward shift in health literacy [9]. The HLS_19_-Q12-HC was validated in a Haitian Creole-speaking immigrant sample in South Florida, and its performance should not be assumed to generalize automatically to Haitian diaspora communities in other national contexts. Additional validation and measurement invariance testing will be needed before cross-national comparisons can be made confidently.

Using European-derived cutpoints, 70.2% of participants were categorized as having inadequate or problematic health literacy, higher than the 47.6% limited health literacy reported in the first European comparative survey [39]. However, this level is similar to rates observed in immigrant groups with limited English proficiency; 68.3% low health literacy limited-English-proficient adults in prior U.S. immigrant-focused studies [9]. This level is also higher than that in some non-immigrant HLS_19_-Q12 samples, suggesting that early post-migration conditions and language discordance may have shifted the distribution downward even when education gradients persist [44].

Several factors likely account for these differences. Our sample was composed largely of recent arrivals (*Md* = 2 years in the U.S.) with substantial socioeconomic constraint (75% reporting household income <$15,000) and completed the survey in Haitian Creole, which may mark language discordance with many U.S. healthcare environments. Immigrant health literacy research consistently shows that limited English proficiency and low health literacy can co-occur and jointly amplify vulnerability, particularly in healthcare access and outcomes [9]. These contextual characteristics make a higher prevalence of difficulty plausible, but prevalence should still be interpreted cautiously because HLS cutpoints were developed for European monitoring contexts and may not be calibrated to immigrant samples. In this study, the most interpretable evidence is not the absolute prevalence but the patterning: a strong education gradient, consistent item-level difficulty concentration in clinical navigation/decision tasks, and meaningful convergence with an established screener.

Item-level findings highlight where difficulties cluster for Haitian immigrants. The most difficult tasks involved accessing and applying clinically consequential information (e.g., mental health information seeking, weighing treatment options, and emergency instructions). These patterns align with the conceptual framing that health literacy reflects an interaction between individual capacities and system complexity, and with models describing pathways through which limited health literacy affects outcomes via access, communication, self-care, and decision-making processes [45]. In contrast, relatively lower difficulty for family/friends’ advice or healthy lifestyle information may reflect the availability of Haitian Creole community information channels in South Florida. This contrast reinforces that health literacy is context-dependent: tasks done within culturally familiar networks may be easier than tasks embedded in time-pressured, English-dominant clinical encounters [22]. At the same time, the HLS_19_-Q12 primarily assesses individual information-management tasks and may only indirectly capture oral or community-mediated forms of health communication that may be especially relevant in Haitian communities [4,22,46].

The apparent paradox of substantial educational attainment alongside high levels of reported difficulty underscores the socioecological nature of health literacy. Even when education confers relative advantages (as demonstrated by the known-groups effect), system demands related to language discordance, insurance processes, referrals, and unfamiliar norms around shared decision-making may shift the entire distribution downward among recent immigrants. This interpretation is consistent with broader evidence that health literacy can be conceptualized both as a clinical “risk” and as a public health “asset,” shaped by environments and institutions, not solely by individual traits [46].

### 4.1. Strengths and Limitations

This study had several strengths. To our knowledge, it is the first general health literacy instrument to be psychometrically validated for Haitian Creole-speaking populations, addressing a critical gap in health equity measurement. The translation and cultural adaptation followed M-POHL guidance, including dual independent forward translations, expert panel review, independent certified back-translation, and cognitive interviews, supporting linguistic clarity and conceptual equivalence prior to field deployment [17]. Psychometric evaluation used a latent-variable CFA/SEM framework guided by COSMIN principles, which aligns with the measurement emphasis of the international HLS_19_ program and limits bias from measurement error in validity estimates [30]. Reliability was supported across complementary indices, and external construct validity was triangulated using two independent sources of evidence, convergent association with the BHLS-HC and known-groups differences by education, providing a more persuasive validity argument than reliance on a single test. The use of MLR estimation was justified by observed response distributions, and sensitivity analyses supported the robustness of conclusions to estimator choice.

Limitations should be interpreted in the context of the study’s embedded design. The validation was conducted within the Cancer Health Disparities Registry (CHDR), a larger ongoing registry study with its own recruitment infrastructure and eligibility criteria; thus, the sample was a convenience cohort rather than a probability-based survey. Although the sample size of 168 was adequate for the confirmatory factor analysis conducted and exceeded the commonly recommended 10:1 participant-to-item ratio for a 12-item scale [26], it remained modest for more complex psychometric procedures, such as measurement invariance testing and more detailed subgroup analyses. The cohort was also predominantly female and concentrated among very recent arrivals, which may have shaped observed distributions and limited direct generalizability to Haitian men and longer-term U.S. residents. Future work should target more gender-balanced sampling and examine measurement invariance across sex and acculturation-related variables. Income and years of U.S. residence were not examined as additional known-groups variables because both showed limited variability in this sample. Prior engagement with the U.S. healthcare system was also not formally assessed, limiting our ability to distinguish health literacy challenges from limited system exposure. Recruitment in South Florida may further limit transferability to Haitian communities in other settings. Cognitive interviews were not audio-recorded, which limited the depth of qualitative documentation. Participants received compensation as part of the broader CHDR protocol; although this reflected study burden, its potential influence on participation in a low-income sample should be acknowledged. Nevertheless, recruitment experience suggested that enrollment remained selective rather than automatic, for example, only about 4 in 10 individuals approached ultimately agreed to participate, indicating that compensation alone was unlikely to fully account for participation. The absence of test–retest reliability data means temporal stability has not been confirmed. Measurement invariance across gender, acculturation level, and national context also remains untested, and predictive validity for outcomes such as healthcare utilization and screening uptake has yet to be established. Finally, European-derived cutpoints were applied provisionally for cross-population comparability; calibration of thresholds for immigrant-origin populations warrants further evaluation. Despite these constraints, the evidence supports the study’s primary objective: establishing a psychometric foundation for the HLS_19_-Q12-HC in an underserved, high-vulnerability population.

### 4.2. Implications for Research and Practice

The HLS_19_-Q12-HC provides a validated tool for quantifying general health literacy among Haitian Creole-speaking adults and can support disparities research, needs assessment, and evaluation of interventions. Future research should test measurement invariance across gender and acculturation-related variables, establish test–retest reliability, and examine predictive validity for healthcare utilization, preventive screening uptake, and outcomes. In practice, the distribution of difficulty and the concentration of challenges in clinical navigation support a “universal precautions” approach—assuming that many patients may experience difficulty understanding and using health information and designing systems accordingly [4]. Language-concordant care, professional interpretation, teach-back, culturally tailored Haitian Creole materials, and patient navigation supports are likely to be high-yield strategies, particularly during early post-migration settlement. The measure may also help identify patients with higher navigation support needs, enabling targeted, higher-intensity navigation rather than a one-size-fits-all approach, even within this high-vulnerability population.

## 5. Conclusions

This study establishes the HLS_19_-Q12-HC as a reliable and valid unidimensional measure of general health literacy among Haitian Creole-speaking immigrants in South Florida. Compared with prior HLS validations, the instrument’s psychometric performance is strong, while the high level of reported difficulty appears consistent with the structural and linguistic barriers faced by recent immigrants. The HLS_19_-Q12-HC can support rigorous monitoring and facilitate targeted, individual and system-level responses to improve communication, navigation, and equity for Haitian immigrant communities.

## Figures and Tables

**Table 1:** Sociodemographic Characteristics and Health Literacy Scores Among Haitian Immigrants (*n* = 168).

Characteristic	*n* (%) or Median [Range]
**Demographics**	
***Age, years***	
Median [Range]	46 [21–87]
18–39	63 (37.5)
40–59	75 (44.6)
≥60	30 (17.9)
***Gender***	
Female	142 (84.5)
Male	26 (15.5)
***Years in the United States***	
Median [Range]	2 [1–50]
1–2	115 (68.5)
3–5	19 (11.3)
6–10	13 (7.7)
>10	21 (12.5)
***Education level***	
≤High school	20 (11.9)
High school graduate/GED	35 (20.8)
Some college/Vocational school	67 (39.9)
College degree or Higher	46 (27.4)
***Household income***	
<$15,000	126 (75.0)
$15,000–$29,999	12 (7.1)
$30,000–$49,999	14 (8.3)
≥$50,000	16 (9.5)
***Health literacy measures***	
HLS_19_-Q12-HC score (0–100)	
Median [Range]	52.3 [0–100]
HLS_19_-Q12-HC category	
Inadequate (0–50)	82 (48.8)
Problematic (>50–66.67)	36 (21.4)
Sufficient (>66.67–83.33)	43 (25.6)
Excellent (>83.33–100)	7 (4.2)
BHLS-HC total score (4–20)	
Median [Range]	10.0 [4–20]
Low HL (4–8)	67 (42.1)
Moderate HL (9–12)	33 (20.8)
High HL (13–20)	59 (37.1)

**Table 2:** Item-Level Descriptive Statistics for the Haitian Creole HLS_19_-Q12 (HLS_19_-Q12-HC) (*n* = 168).

Item	“On a Scale from Very Easy to Very Difficult, How Easy Would You Say It Is…”	Mean (SD)	% Difficult/Very Difficult	*r* (Item–Total)
1	…to find out where to get professional help when you are ill? [instructions: such as doctor, nurse, pharmacist, psychologist]	2.48 (0.77)	49.4	0.687
2	…to understand information about what to do in a medical emergency?	2.42 (0.80)	56.0	0.733
3	…to judge the advantages and disadvantages of different treatment options?	2.26 (0.90)	56.0	0.712
4	…to act on advice from your doctor or pharmacist?	2.46 (0.73)	52.4	0.785
5	…to find information on how to handle mental health problems? [Instruction: stress, depression or anxiety]	2.29 (0.75)	63.1	0.685
6	…to understand information about recommended health screenings or examinations? [Instructions: e.g., colorectal cancer screening, blood sugar test]	2.42 (0.73)	54.2	0.774
7	…to judge if information on unhealthy habits, such as smoking, low physical activity or drinking too much alcohol, are reliable?	2.56 (0.73)	44.0	0.804
8	…to decide how you can protect yourself from illness using information from the mass media? [Instructions: e.g., Newspapers, TV or Internet]	2.49 (0.70)	49.4	0.725
9	…to find information on healthy lifestyles such as physical exercise, healthy food or nutrition?	2.57 (0.72)	44.6	0.780
10	…to understand advice concerning your health from family or friends?	2.60 (0.68)	39.9	0.689
11	…to judge how your housing conditions may affect your health and well-being?	2.53 (0.74)	46.4	0.818
12	…to make decisions to improve your health and well-being?	2.51 (0.73)	48.8	0.798
	**Overall scale**	**2.47 (0.74)**	**50.3**	**0.685–0.818**

**Table 3:** Internal Consistency and Convergent Reliability Indices for the Haitian Creole HLS_19_-Q12 (HLS_19_-Q12-HC).

Reliability/Validity Index	Estimate
McDonald’s omega (ω)	0.949
Cronbach’s alpha (α)	0.944
Average Variance Extracted (AVE)	0.584

**Table 4:** Confirmatory Factor Analysis Model Fit Comparison for Alternative Factor Structures of the HLS_19_-Q12-HC (*n* = 168).

Model Specification	χ^2^ (df)	*p*-Value	CFI	TLI	RMSEA (90% CI)	SRMR	χ^2^/df	ΔCFI
1-factor (unidimensional)	91.85 (54)	0.001	0.951	0.944	0.065 (0.044–0.084)	0.048	1.70	-
2-factor (theory-based)	69.98 (53)	0.059	0.978	0.974	0.044 (0.000–0.067)	0.041	1.32	0.027
3-factor (HLS-EU domains)	72.44 (51)	0.026	0.972	0.967	0.050 (0.020–0.072)	0.043	1.42	0.021

**Table 5:** Standardized Factor Loadings and Item Performance for the Unidimensional HLS_19_-Q12-HC Model (*n* = 168).

Item	“On a Scale from Very Easy to Very Difficult, How Easy Would You Say It Is…”	*λ* (Std.)	*SE*	*p*	*R* ^2^
1	…to find out where to get professional help when you are ill? [instructions: such as doctor, nurse, pharmacist, psychologist]	0.690	0.048	<0.001	0.476
2	…to understand information about what to do in a medical emergency?	0.732	0.039	<0.001	0.535
3	…to judge the advantages and disadvantages of different treatment options?	0.725	0.037	<0.001	0.525
4	…to act on advice from your doctor or pharmacist?	0.808	0.032	<0.001	0.653
5	…to find information on how to handle mental health problems? [Instruction: stress, depression or anxiety]	0.699	0.045	<0.001	0.488
6	…to understand information about recommended health screenings or examinations? [Instructions: e.g., colorectal cancer screening, blood sugar test]	0.790	0.038	<0.001	0.624
7	…to judge if information on unhealthy habits, such as smoking, low physical activity or drinking too much alcohol, are reliable?	0.834	0.033	<0.001	0.696
8	…to decide how you can protect yourself from illness using information from the mass media? [Instructions: e.g., Newspapers, TV or Internet]	0.764	0.051	<0.001	0.584
9	…to find information on healthy lifestyles such as physical exercise, healthy food or nutrition?	0.809	0.048	<0.001	0.655
10	…to understand advice concerning your health from family or friends?	0.724	0.069	<0.001	0.524
11	…to judge how your housing conditions may affect your health and well-being?	0.851	0.037	<0.001	0.724
12	…to make decisions to improve your health and well-being?	0.836	0.031	<0.001	0.699

**Table 6:** Evidence of Convergent and Known-Group Construct Validity of the HLS_19_-Q12-HC from Structural Equation Modeling Analyses.

Validity Type	External Measure/Predictor	*n*	Standardized Estimate (*β*)	*SE*	*p*-Value	95% CI	*R* ^2^
Convergent	BHLS-HC total score	159	0.518	0.058	<0.001	[0.405, 0.631]	0.268
Known-groups	Education (4 levels)	168	0.463	0.074	<0.001	[0.318, 0.608]	—

## Data Availability

The original contributions presented in this study are included in the article/Supplementary Materials. Further inquiries can be directed to the corresponding author.

## Supplementary Materials

The following supporting information can be downloaded at: https://www.mdpi.com/article/10.3390/ijerph23050554/s1, Table S1. Brief Health Literacy Screen—Haitian Creole Version (BHLS-HC): Descriptive and Reliability Statistics (*n* = 159); Table S2. Response Distribution for HLS_19_-Q12-HC Items (*n* = 168); Table S3. Subscale Reliabilities and Inter-Factor Correlations for Alternative Multidimensional Factor Structures of the HLS_19_-Q12-HC (*n* = 168); Table S4. Post-Hoc Pairwise Comparisons of Mean Health Literacy Scores Across Education Levels Using Tukey’s Honestly Significant Difference Test (*n* = 168); Table S5. Changes to the HLS_19_-Q12 when comparing English and Haitian Creole versions; Table S6. Comparison of Unidimensional Model Results Across MLR and WLSMV Estimation Methods (*n* = 168).

## Abbreviations

The following abbreviations are used in this manuscript:

AVE	Average Variance Extracted
BHLS	Brief Health Literacy Screen
BHLS-HC	Haitian Creole Brief Health Literacy Screen
CFA	Confirmatory Factor Analysis
CFI	Comparative Fit Index
CHDR	Cancer Health Disparities Registry
CI	Confidence Interval
COSMIN	Consensus-based Standards for the Selection of Health Measurement Instruments
GED	General Educational Development
HLS_19_-Q12	12-item Health Literacy Survey 2019
HLS_19_-Q12-HC	Haitian Creole version of the 12-item Health Literacy Survey 2019
HLS-EU	European Health Literacy Survey
IRB	Institutional Review Board
M-POHL	Action Network on Measuring Population and Organizational Health Literacy
MLR	Maximum Likelihood with Robust Standard Errors
RMSEA	Root Mean Square Error of Approximation
SD	Standard Deviation
SEM	Structural Equation Modeling
SRMR	Standardized Root Mean Square Residual
TLI	Tucker–Lewis Index
U.S.	United States
WLSMV	Weighted Least Squares Mean and Variance Adjusted
