# Supplementary material for: Measuring General Health Literacy in Haitian Immigrant Adults: Validation of the HLS19-Q12 Instrument in Haitian Creole
Source: Int J Environ Res Public Health. 2026 Apr 25;23(5):554. doi: 10.3390/ijerph23050554 (PMC13205792; doi:10.3390/ijerph23050554)
Supplement: Supplementary file 1 [file ijerph-23-00554-s001.zip › ijerph-4263297-supplementary.pdf]

Supplementary Materials

Table S1. Brief Health Literacy Screen—Haitian Creole Version (BHLS-HC): Descriptive and Reliability Statistics (N = 159).

S1a. Scale-level summary

Measure	Value
Cronbach's alpha (α)	0.87
McDonald's omega (ω)	0.88
Total score, mean (SD)	10.0 (4.2)
Total score, median [range]	10.0 [4–20]

S1b. Item-level statistics

Item (short label)	Mean (SD)	Item–total r
Confidence filling out medical forms	2.8 (1.3)	0.78
Need help reading hospital materials	2.6 (1.2)	0.81
Problems learning about medical condition	2.9 (1.1)	0.75
Confidence understanding written information	2.7 (1.3)	0.82

Table S2. Response Distribution for HLS$_{19}$-Q12-HC Items (N = 168)

Item	Item content	Very difficult n (%)	Difficult n (%)	Easy n (%)	Very easy n (%)
1	…to find out where to get professional help when you are ill? [instructions: such as doctor, nurse, pharmacist, psychologist]	38 (22.6)	56 (33.3)	62 (36.9)	10 (6.0)
2	…to understand information about what to do in a medical emergency?	32 (19.0)	51 (30.4)	71 (42.3)	12 (7.1)
3	…to judge the advantages and disadvantages of different treatment options?	44 (26.2)	62 (36.9)	54 (32.1)	6 (3.6)
4	…to act on advice from your doctor or pharmacist?	28 (16.7)	48 (28.6)	78 (46.4)	12 (7.1)
5	…to find information on how to handle mental health problems? [Instruction: stress, depression or anxiety]	41 (24.4)	56 (33.3)	60 (35.7)	9 (5.4)
6	…to understand information about recommended health screenings or examinations? [Instructions: e.g. colorectal cancer screening, blood sugar test]	30 (17.9)	45 (26.8)	79 (47.0)	12 (7.1)
7	...to judge if information on unhealthy habits, such as smoking, low physical activity or drinking too much alcohol, are reliable?	22 (13.1)	45 (26.8)	86 (51.2)	13 (7.7)
8	…to decide how you can protect yourself from illness using information from the mass media? [Instructions: e.g. Newspapers, TV or Internet]	26 (15.5)	52 (31.0)	76 (45.2)	12 (7.1)
9	…to find information on healthy life styles such as physical exercise, healthy food or nutrition?	18 (10.7)	42 (25.0)	92 (54.8)	14 (8.3)
10	…to understand advice concerning your health from family or friends?	24 (14.3)	46 (27.4)	84 (50.0)	12 (7.1)
11	...to judge how your housing conditions may affect your health and well-being?	20 (11.9)	38 (22.6)	94 (56.0)	14 (8.3)
12	…to make decisions to improve your health and well-being?	22 (13.1)	44 (26.2)	88 (52.4)	12 (7.1)

Table S3. Subscale Reliabilities and Inter-Factor Correlations for Alternative Multidimensional Factor Structures of the HLS19-Q12-HC (N = 168)

S3a. Two-factor model

Factor	Item numbers	Items (n)	Cronbach's α	McDonald's ω	Inter-factor correlation
F1: Clinical/Healthcare Navigation	1–7	7	0.910	0.912	
F2: Health Promotion/Everyday HL	8–12	5	0.907	0.910	r (F1, F2) = 0.91*

S3b. Three-factor model (HLS-EU domains)

Factor	Item numbers	Items (n)	Cronbach's α	McDonald's ω	Inter-factor correlations
F1: Healthcare	1–4	4	0.857	0.860	r (F1, F2) = 0.886*; r(F1, F3) = 0.912*
F2: Disease Prevention	5–8	4	0.862	0.866	r (F2, F3) = 0.942*
F3: Health Promotion	9–12	4	0.897	0.901	—

*p < .01 for all factor correlations

Table S4. Post-Hoc Pairwise Comparisons of Mean Health Literacy Scores Across Education Levels Using Tukey's Honestly Significant Difference Test (N = 168)

Education comparison	Group 1 mean (SD)	Group 2 mean (SD)	Mean difference	95% CI	p value[a]	Cohen's d[b]
≤High school vs High school graduate/GED	22.4 (35.3)	32.7 (37.6)	-10.3	[-28.4, 7.8]	.382	-0.28
≤High school vs Some college/vocational	22.4 (35.3)	52.0 (35.0)	-29.6	[-47.2, -12.0]	<.001	-0.84
≤High school vs College degree or higher	22.4 (35.3)	70.8 (31.4)	-48.4	[-65.2, -31.6]	<.001	-1.46
High school graduate/GED vs Some college/vocational	32.7 (37.6)	52.0 (35.0)	-19.3	[-32.6, -6.0]	<.001	-0.54
High school graduate/GED vs College degree or higher	32.7 (37.6)	70.8 (31.4)	-38.1	[-51.4, -24.8]	<.001	-1.08
Some college/vocational vs College degree or higher	52.0 (35.0)	70.8 (31.4)	-18.8	[-30.2, -7.4]	<.001	-0.56

[a] Tukey's honestly significant difference (HSD) adjusted p values.

[b] Cohen's d reflects standardized mean differences (Group 1 − Group 2); negative values indicate higher scores in Group 2.

Table S5. Changes to the HLS19 -Q12 when comparing English and Haitian Creole versions

Item	Original (English)	Back-translation (English)	Notes
1	…to find out where to get professional help when you are ill? [instructions: such as doctor, nurse, pharmacist, psychologist]	…to know where to go or whom to consult when you are sick; such as a doctor, nurse, pharmacist, or psychologist?	**Same meaning.** Minor wording shift: "find out where to get" → "know where to go/whom to consult."
2	…to understand information about what to do in a medical emergency?	…to understand information about what to do in an emergency when someone is sick or injured?	**Same meaning.** minor changes: specifies "when someone is sick or injured," which clarifies but does not change meaning.
3	…to judge the advantages and disadvantages of different treatment options?	…to compare different treatments (such as taking medication or having surgery) to understand what is best or most risky for you?	**Same meaning.** "Judge advantages/disadvantages" → "compare treatments" with added examples. Slight shift toward personal risk/benefit framing ("best or most risky for you"), but overall construct retained.

4	…to act on advice from your doctor or pharmacist?	…to follow your doctor's or pharmacist's advice. For example, on how to take medication or change your habits?	**Same meaning** "Act on advice" → "follow advice". Added concrete examples (medication, lifestyle change).
5	…to find information on how to handle mental health problems? [Instruction: stress, depression or anxiety]	…to find information on what to do about mental health issues (stress, sadness, anxiety, or depression)?	**Same meaning.** "Handle mental health problems" reframed into specific conditions.
6	…to understand information about recommended health screenings or examinations? [Instructions: e.g. colorectal cancer screening, blood sugar test]	…to understand why and when you are advised to have health tests such as blood sugar tests or cancer screenings (pap test, mammography)?	**Same meaning.** Added "why and when" (timing and rationale). Examples expanded/adjusted (Pap test, mammography instead of colorectal screening). Concept unchanged.
7	...to judge if information on unhealthy habits, such as smoking, low physical activity or drinking too much alcohol, are reliable?	…to judge whether information about unhealthy behaviors (such as smoking, drinking too much or not being physically active) is reliable?	**Same meaning.** Minor wording adjustments: "low physical activity" → "not being physically active"; "drinking too much alcohol" → "drinking too much"
8	…to decide how you can protect yourself from illness using information from the mass media? [Instructions: e.g. Newspapers, TV or Internet]	…to use information from radio, television, or the internet to learn how to protect yourself from illness?	**Same meaning.** "Decide how to protect yourself" → "use information to learn how to protect yourself". Media examples slightly modified (adds radio, removes newspapers). Core intent retained.
9	…to find information on healthy life styles such as physical exercise, healthy food or nutrition?	…to find information about how to eat well, exercise, and live a healthy lifestyle?	**Same meaning.** Smoother phrasing; "healthy food/nutrition" → "eat well."
10	…to understand advice concerning your health from family or friends?	…to understand advice your family or friends give you about health?	**Same meaning.**
11	...to judge how your housing conditions may affect your health and well-being?	…to judge how things such as the air you breathe, the water you drink, noise, or cleanliness in the home can affect your health and well-being?	**Same meaning.** Minor changes to add concrete examples
12	…to make decisions to improve your health and well-being?	…to make decisions to stay healthy (eating better, exercising, or resting) and to take better care of yourself?	**Same meaning.** Adds examples (diet, exercise, rest). "Improve health and well-being" → "stay healthy / take better care of yourself."

Table S6. Comparison of Unidimensional Model Results Across MLR and WLSMV Estimation Methods (N = 168)

Metric	MLR Estimation	WLSMV Estimation
**Model Fit**		
χ^2 (df)	91.85 (54)	253.13 (54)
p-value	.001	<.001
CFI	0.951	0.971
TLI	0.944	0.965
RMSEA (90% CI)	0.065 (0.044–0.084)	0.148 (0.121–0.176)
SRMR/WRMR	0.048	0.058
**Factor Loadings (range)**	0.690–0.851	0.768–0.895
**Reliability**		
McDonald's ω	0.949	0.965
Cronbach's α	0.944	0.961
